# The use of learning collaboratives to support integrated mental health and opioid use disorder services: lessons from the HEALing Communities Study

**DOI:** 10.3389/fpsyg.2026.1698589

**Published:** 2026-03-06

**Authors:** Victoria R. Votaw, Maria Rudorf, Ileana Dragoi, Donna Beers, Vanessa Loukas, Sara M. Roberts, R. Kathryn McHugh, Roger D. Weiss

**Affiliations:** 1 Division of Alcohol, Drugs, and Addiction, McLean Hospital, Belmont, MA, United States; 2 Department of Psychiatry, Harvard Medical School, Boston, MA, United States; 3 Grayken Center for Addiction, Section of General Internal Medicine, Boston Medical Center, Boston, MA, United States; 4 School of Medicine, Case Western Reserve University, Cleveland, OH, United States

**Keywords:** co-occurring disorders, learning collaboratives, opioid use disorder, overdose, training

## Abstract

Mental health concerns (e.g., psychiatric disorders, traumatic experiences, suicidal ideation) are common among those with opioid use disorder (OUD) and are associated with an increased risk of overdose. Yet, few people with OUD receive integrated treatment for psychiatric disorders or other concerns, underscoring the need to reduce barriers to integrated care, such as a lack of knowledge about mental health conditions among frontline workers and providers. In this Community Case Study, we describe an effort at a single site (Massachusetts) of the HEALing Communities Study (HCS) to address barriers to integrated care through Learning Collaboratives (LCs), a method for large-scale dissemination of evidence-based practices. The HCS was a parallel-group, community-engaged, cluster-randomized trial conducted across four states/sites from January 2020 to December 2023 to reduce fatal opioid-related overdoses. LC sessions were offered to partners in HCS communities (e.g., frontline workers and providers, such as peer recovery coaches, social workers, psychiatrists) to support overdose reduction strategies. The HCS Massachusetts site implemented an LC series focused on mental health and OUD, including didactic presentations emphasizing concrete take-home messages and intervention strategies, case presentations, and interactive discussions. Provider participants from various professional backgrounds rated the LC sessions as helpful and useful, highlighting the feasibility of this method for disseminating information on evidence-based practices to address OUD and co-occurring mental health concerns. We also discuss challenges and lessons learned by the planning team to inform future LCs that address integrated care for OUD and mental health concerns.

## Introduction

More than half of adults with opioid use disorder (OUD) have a co-occurring psychiatric disorder (Santo et al., 2022). Mental health concerns potentially requiring assessment and intervention among individuals with OUD also extend beyond formal psychiatric diagnoses. For example, over 70% of individuals with OUD have experienced at least one traumatic event, including childhood trauma (Rodríguez et al., 2024; Jeffries-Baxter et al., 2024). Of particular concern are findings that rates of suicidal ideation, planning, and attempts in this population are more than six times higher than those seen in the general population (Streck et al., 2022).

These mental health concerns among those with opioid misuse and OUD might contribute to the ongoing opioid overdose crisis. A recent systematic review reported consistent associations between psychiatric disorders and opioid overdoses, including across populations (e.g., general population, people who use prescription opioids, youth), research designs (e.g., retrospective vs. prospective), and overdose type (e.g., fatal vs. non-fatal; van Draanen et al., 2022). Multiple mechanisms likely underlie the relationship between psychiatric disorders and opioid overdose, although causal pathways remain largely unexplored (van Draanen et al., 2022). However, emerging evidence among participants who experienced a non-fatal opioid overdose suggests that thoughts of death or a desire to die are common preceding factors in overdose events (Connery et al., 2019, 2022). These findings indicate that suicidal motivations might represent a critical link between psychiatric disorders and the risk of opioid overdose. Taken together, there is a need for integrated approaches that assess and treat psychiatric symptoms, including suicidal ideation, alongside evidence-based OUD treatment and overdose prevention strategies (e.g., naloxone distribution; Hawk et al., 2015).

Despite the high rates of mental health concerns among those with OUD and their impact on overdose risk, only a quarter of those with co-occurring OUD and psychiatric disorders report receipt of concurrent substance use disorder (SUD) and mental health treatment (Novak et al., 2019; Jones and McCance-Katz, 2019). Furthermore, only one-third of opioid treatment programs offer a comprehensive mental health assessment (Substance Abuse and Mental Health Services Administration, 2021), and less than half offer mental health services alongside other evidence-based interventions, including medications for OUD and contingency management (Park et al., 2024). Among individuals with co-occurring OUD and psychiatric disorders, the primary obstacles to mental health and substance use treatment include affordability, treatment access, and stigma (Novak et al., 2019). Provider- and systems-level barriers to integrated treatment include provider shortages, limited time and resources (e.g., inadequate time to deliver more than case management, limited resources for referral or escalating care), prioritization of substance use over mental health concerns in evaluations, and lack of training regarding the treatment of co-occurring conditions (Snell-Rood et al., 2021; Winiker et al., 2023). Broader systemic barriers, such as gaps in the continuum of care, limited availability of external mental health services, difficulties with insurance coverage, and long waitlists, compound these provider-level challenges (Snell-Rood et al., 2021; Winiker et al., 2023). There is an urgent need to reduce these provider- and systems-level barriers and increase the availability of integrated services for mental health concerns and OUD.

Learning collaboratives (LCs) represent an innovative and scalable approach to addressing provider-level barriers to integrated treatment. LCs serve as forums for disseminating evidence-based practices via shared learning among providers from multiple organizations and ongoing professional networks and support (Nadeem et al., 2014; Gotham et al., 2023). LCs can include various elements (though not all are utilized simultaneously in every LC), such as learning sessions, multidisciplinary quality improvement teams, plan-do-study-act (PDSA) cycles for quality improvement, and quality improvement team calls (Nadeem et al., 2014). As such, LCs typically involve both knowledge dissemination (primarily through learning sessions and shared learning) and implementation (through quality improvement elements). Learning sessions, the most common elements used in LCs, can vary in frequency, duration, and length, ranging from 90-min to three-day-long sessions, delivered one to four times (Nadeem et al., 2014). While these largely comprise shared, peer-based learning, they typically also include some didactic information (Nadeem et al., 2014). This approach has been applied to mental health care for various purposes, such as increasing the use of an evidence-based depression assessment in psychiatric practices (Duffy et al., 2008), disseminating cognitive processing therapy to community settings (LoSavio et al., 2019), and improving time to treatment and retention in SUD treatment (Gustafson et al., 2013). Overall, LCs represent a promising approach to disseminating evidence-based, integrated care practices to OUD treatment providers in community settings and to other frontline workers (e.g., harm reduction workers) who may have limited knowledge of behavioral health care for co-occurring conditions.

In this Community Case Study, we describe an effort within a single site (Massachusetts; MA) of the HEALing Communities Study (HCS) (The HEALing Communities Study Consortium, 2024; Walsh et al., 2020; Winhusen et al., 2020) to address barriers to mental health care during OUD treatment through LCs. The HCS was a multi-site, parallel-group, community-engaged, cluster-randomized trial conducted across four sites/states from January 2020 to December 2023 to reduce fatal opioid-related overdoses (The HEALing Communities Study Consortium, 2024; Walsh et al., 2020; Winhusen et al., 2020). The HCS used LCs (in the form of standalone sessions and/or series) to disseminate information on evidence-based overdose prevention strategies through didactics, case examples, and discussions among attendees who were primarily frontline workers (e.g., harm reduction service workers, peer recovery coaches), treatment providers, and clinicians from different teams/organizations across study states/sites, thus promoting widespread dissemination of information. Of note, these efforts relied entirely on a series of learning sessions rather than other LC elements (Nadeem et al., 2014). The MA site facilitated a Mental Health and OUD LC series, which is described herein. This LC series aimed to increase knowledge of mental health concerns that commonly co-occur with OUD and interventions targeting these conditions. We also present attendee data on the utility of these LC sessions and describe the planning team's perceptions of challenges encountered and lessons learned in implementing the LC series to inform future efforts.

## Context

### Overview of the HEALing Communities Study and learning collaborative sessions

Details on the HCS have been extensively reported elsewhere (The HEALing Communities Study Consortium, 2024; Walsh et al., 2020; Winhusen et al., 2020). Briefly, the four HCS sites were in MA, Kentucky, New York, and Ohio, spanning 67 communities. Inclusion criteria for communities in the HCS included willingness to select and implement evidence-based strategies guided by the Opioid-overdose Reduction Continuum of Care Approach (ORCCA; Winhusen et al., 2020). In addition, each HCS state/site was required to have (1) >150 opioid-related overdose fatalities and (2) >25 opioid-related overdose fatalities per 100,000 people in 2016. Additionally, >30% of selected communities were rural. HCS communities were treated as clusters and were allocated to the intervention during the first 2 years (Wave 1; January 2020-June 2022) or waitlist control after a 2-year delay (Wave 2; July 2022-December 2023).

Intervention strategies within the ORCCA focused on three areas: (1) overdose education and naloxone distribution, (2) effective delivery of medications for OUD, and (3) safer opioid prescribing (Walsh et al., 2020). In implementing the ORCCA intervention strategies, the HCS enhanced partnerships between community members and leaders, including medical and behavioral health providers, the criminal-legal sector, frontline workers (e.g., harm reduction service workers, peer recovery specialists), treatment providers (e.g., psychiatrists, therapists), community organizations, and people who use drugs. In addition, Training and Technical Assistance (TTA) was provided throughout the study to support communities in implementing evidence-based overdose reduction strategies and included Trainings (i.e., didactic presentations, such as trainings on levels of care), Technical Assistance (TA; i.e., assisting with procedures and resources, such as setting up billing for a new service), and LC sessions (Winhusen et al., 2020).

The HCS LC sessions were primarily facilitated virtually to allow for statewide participation. Session attendance was voluntary and open to a range of community service workers and providers in the field of substance use and mental health. Sessions were 1–2 h, with a facilitated didactic and/or presentation followed by an open discussion to answer questions and facilitate learning from provider peers; when applicable, sessions also included case presentations by community participants. LC topics (standalone or series) were primarily determined in partnership with community participants and research staff to support the adoption and implementation of ORCCA interventions. As such, topics varied across sites and spanned several categories, including advocacy, clinical care, housing, mental health, harm reduction, peer support, and racial and social justice, among others.

### Target setting and population: Massachusetts site

MA was the only HCS site to offer a Mental Health and OUD LC series. This site included 16 municipalities or clusters of rural municipalities (Walsh et al., 2020), and preferred communities with an office-based addiction treatment (OBAT) program and pre-existing substance use coalitions. The primary target to attend the Mental Health and OUD LC series was OUD frontline workers and providers within HCS communities. Table 1 presents an overview of attendees' professional backgrounds, collected during LC registration. The most commonly reported professional backgrounds were in community services, such as community outreach coordinators and housing services, and in patient care, such as nurses, psychiatrists, counselors, social workers, and case managers. Otherwise, there was representation from those in recovery/prevention services (e.g., harm reduction specialists, peer recovery coaches), healthcare management/administration (e.g., organization administrative coordinators, medical directors), and law enforcement/security (e.g., probation officers, inmate service directors). Some attendees also reported research and academic roles, though these were largely employees at HCS research sites rather than community members.

**Table 1:** Overview of professions from attendees of the Mental Health and Opioid Use Disorder Learning Collaborative series.

**Wave**	**Profession sectors**
Wave 1 (*n* = 423)	• Patient care (*n* = 104) • Community services (*n* = 99) • Recovery/prevention services (*n* = 36) • Healthcare management/administration (*n* = 50) • Research/academic roles (*n* = 35) • Law enforcement/security (*n* = 14) • Other (*n* = 30) • Not reported (*n* = 55)
Wave 2 (*n* = 370)	• Patient care (*n* = 66) • Community services (*n* = 143) • Recovery/prevention services (*n* = 33) • Healthcare management/administration (*n* = 47) • Research/academic roles (*n* = 28) • Law enforcement/security (*n* = 11) • Other (*n* = 23) • Not reported (*n* = 19)
Community X (*n* = 237)	• Patient care (*n* = 89) • Community services (*n* = 36) • Recovery/prevention services (*n* = 17) • Healthcare management/administration (*n* = 16) • Research/academic roles (*n* = 18) • Law enforcement/security (*n* = 3) • Other (*n* = 12) • Not reported (*n* = 46)

^a^Participants who attended sessions multiple times were counted each time they attended, given certain aspects of participant registration precluded the feasibility of only including each participant once (e.g., attendees did not include their name or full name). In addition, HCS staff, non-HCS staff, host(s), presenter(s), and facilitator(s) are all included.

^b^Profession sectors include the following roles: Patient Care (nurses, clinicians, psychiatrists, social workers); Community Services (community outreach/coordinators); Recovery/Prevention Services (harm reduction specialists, peer recovery coaches); Healthcare Management/Administration (medical directors, CMOs, administrative coordinators); Research/Academic Roles (associate professors, research assistants, division chiefs, interns); and Law Enforcement/Security (security personnel, monitors, probation officers).

## Key programmatic elements

### Planning the Mental Health and Opioid Use Disorder Learning Collaboratives

The team responsible for planning the Mental Health and OUD LC series included HCS staff and investigators at the primary MA research site (Boston Medical Center; BMC) and a subcontracted research site (McLean Hospital). The TTA Management Specialist at the MA site (MR) served as the primary organizer of all site LCs and, therefore, spearheaded the planning process for the series (e.g., recruiting and scheduling community speakers, communicating with community members about upcoming sessions). To assist with session facilitation and connection with mental health specialists in communities, additional support came from a nurse practitioner at BMC with expertise in co-occurring substance use and psychiatric disorders (VL). In addition, clinician-scientists at McLean Hospital (RKM, a clinical psychologist, and RDW, an addiction psychiatrist) served as content specialists in co-occurring OUD and psychiatric disorders. Content specialists were critical to identifying additional specialists, primarily through their professional networks, who facilitated didactic presentations (described further below).

The team met quarterly to plan the LC series topics and structure. At these meetings, the team reviewed topics of interest to community members based on responses to post-session feedback forms (see Table 4 for examples). This group selected topics based on community interest, potential impact on patient care (e.g., common mental health conditions), and applicability to opioid overdose prevention; see Table 2 for topics delivered. The organizational team primarily leveraged their professional networks to identify and invite content specialists to present didactics. HCS staff then sent a general email to coalition members and community partners announcing the upcoming topic and requesting volunteers to present a case. In addition, community members who consistently attended the LC series were directly recruited to present cases. Case presenters were not compensated.

**Table 2:** Overview of mental health and opioid use disorder learning collaborative sessions, content, and attendance.

**Topic**	**Didactic content**	**Wave 1**		**Wave 2**		**Community X**	
		**Case example included (yes/no)**	**Total attendance/non-HCS attendance^a^ (# of feedback responses)**	**Case example included (Yes/No)**	**Total attendance/non-HCS attendance^a^ (# of feedback responses)**	**Case example included (yes/no)**	**Total attendance/non-HCS attendance^a^ (# of feedback responses)**
Posttraumatic stress disorder (PTSD)	A presentation on the intersection of PTSD and OUD.	Yes	32/16 (4)	No	22/12 (NR)	Yes	35/24 (9)
Bipolar disorder	A presentation on the diagnosis and management of bipolar and OUD.	Yes	22/13 (7)	No	17/12 (3)	No^b^	64/49 (13)
Sleep	A presentation on the intersection of sleep and OUD.	Yes	20/12 (4)	No	21/13 (5)	NA	NA
Motivational interviewing (MI)	A presentation on specific MI tools for effective conversations about change.	Yes	41/31 (6)	No	26/21 (8)	NA	NA
Suicide	Presentation(s) on suicide prevention and OUD. Wave 1 had a two-part series, and wave 2 had a single presentation.	No^b^	Part 1: 37/29 (4) Part 2: 24/18 (3)	No	36/26 (NR)	NA	NA
Alcohol use disorder (AUD)	A presentation on the intersection of AUD and OUD.	Yes	52/43 (11)	No	32/21 (8)	NA	NA
Medications for AUD	A presentation on medications for AUD in people with OUD.	NA	NA	No	19/15 (2)	NA	NA
De-escalation	A presentation on de-escalation techniques for people with OUD.	Yes	51/42 (11)	No	59/50 (1)	NA	NA
Anxiety disorders	A presentation on the intersection of anxiety and OUD.	No	37/30 (13)	No	28/15 (NR)	NA	NA
Trauma-informed care (TIC)	A presentation on providing trauma-informed care for patients with OUD.	Yes	33/27 (2)	NA	NA	No	25/14 (1)
Civil commitment	A presentation on section 12 & 35 civil commitment criteria.	No	40/30 (7)	No	23/12 (3)	NA	NA
Treatments for stimulant use disorders	A presentation on stimulant use, withdrawal, and behavioral management.	No	34/28 (6)	Yes	24/19 (6)	NA	NA
Personality disorders	A presentation on the intersection of personality disorders and OUD.	NA	NA	No	27/29 (NR)	Yes	51/40 (6)
ADHD	A presentation on the intersection of ADHD and OUD.	NA	NA	No	22/12 (2)	NA	NA
Depression	A presentation on the intersection of depression and OUD.	NA	NA	No	14/8 (2)	Yes	34/24 (3)
Benzodiazepines	A presentation on the topic of prescribing benzodiazepines for patients with SUD.	NA	NA	NA	NA	No	28/17 (2)
Final session roundtable topics	A series of roundtable, high-level summary presentation on key mental health and OUD topics previously covered.	NA	NA	No	19/7 (NR)	NA	NA

^a^Total Attendance includes all HCS staff, host(s), presenter(s), and facilitator(s).

^b^These two sessions were defined as “trainings” instead of LCs because they focused less on participant engagement, including discussion and case presentations, than LCs. Nevertheless, they were part of the LC series and are therefore included in this description.

NA, not applicable indicates a certain topic was not delivered to a given wave; NR, no responses were provided to feedback surveys.

### Delivery of the Mental Health and Opioid Use Disorder Learning Collaboratives

The Mental Health and OUD LCs were delivered monthly during Waves 1 and 2. During Wave 1, 11 LCs were offered from June 2021 to June 2022, and these sessions were exclusive to Wave 1 community members. During Wave 2, 15 LCs were offered from September 2022 to December 2023, and these sessions were open to communities across both waves. Initially, LCs were listed as an option for community coalitions to select for delivery, but as planning for HCS progressed, HCS investigators decided to provide LCs to all communities. Before this change, one Wave 1 community (hereafter, Community X) specifically requested the Mental Health and OUD LC series for its community. As such, Community X received a separate Mental Health and OUD LC series comprising of six sessions from September 2020 to May 2021.

Table 2 presents an overview of LC sessions across all waves, including selected topics, didactic content overviews, and whether case examples were included. Generally, sessions were 1 h and included a didactic portion facilitated by a content specialist, a case example presented by a community partner who was a frontline worker or provider, and a question-and-answer and discussion session to promote peer-based, shared learning among attendees. Time spent on each component depended on the topic and the availability of a case presentation. Didactic sessions typically included evidence-based information on the prevalence of specific mental health concerns among individuals with OUD, along with a brief overview of risk factors and potential reasons (i.e., mechanisms) for a given co-occurrence. Didactics also included information on recognizing mental health conditions, therapeutic strategies that could be implemented by providers of diverse professional backgrounds, other intervention options potentially requiring specialist referrals, information on health equity (e.g., racial and gender health disparities in the data), and relevant resources (e.g., National Sexual Assault Crisis Hotline, mental health apps). Case examples by community partners included patient demographics, diagnoses, substance use history, other relevant history (e.g., trauma, overdose), psychosocial stressors/challenges, strengths and protective factors, societal/structural barriers to treatment, patient goals, and discussion questions, all while ensuring confidentiality. If we could not secure a community partner for a case presentation, the content specialist would share a case, or the shared, peer-led discussion would be extended, providing attendees with time to discuss questions or cases that may be relevant, without the need for formal preparation.

As an illustrative example, the didactic for the session on *Sleep and OUD* provided an overview of prevalence rates of sleep disturbances in OUD; disparities in sleep health and health care; mechanistic relationships between sleep disruption and overdose (e.g., increased risk of co-using opioids and benzodiazepines); common signs of sleep disorders; medications and cognitive-behavioral therapy for insomnia; concrete strategies to improve sleep (e.g., minimizing light exposure, stimulus control); and resources for managing sleep in people with SUDs. The corresponding case for this session was co-presented by a registered nurse and a bachelor's-level alcohol and drug counselor at a community addiction treatment program that offers residential and outpatient services. The presenters described a middle-aged white female who had been receiving buprenorphine treatment for 7 years. This patient was chronically unhoused, reported anxiety, depression, and polysubstance use (i.e., opioids and cocaine), and had a history of childhood sexual abuse and four overdoses. Concerning sleep problems, the patient reported anxiety and hypervigilance at bedtime, given that her unstable housing provided challenges to finding a safe place to sleep, including the inability to safely store medications (e.g., buprenorphine), and the negative impacts of buprenorphine on sleep. The subsequent discussion among attendees focused on safe medication storage for patients who lack safe and stable housing, and when patients should take buprenorphine to avoid sleep disturbances.

This example is illustrative of the cases commonly presented in the LC sessions, which often included multiple psychiatric and medical conditions, housing insecurity, and other significant social and environmental stressors. Accordingly, a recurring focus of the case discussions was on applying evidence-based treatment principles within highly challenging contexts.

### Participant attendance and feedback from the Mental Health and Opioid Use Disorder Learning Collaboratives

In the final minutes of an LC session, an optional feedback survey was shared in the Zoom meeting chat for attendees to respond to. The survey link was also shared after the session in a follow-up email to all attendees. The survey included items (primarily Likert-scale; see Table 3) assessing the perceived helpfulness and relevance of the LC, the usefulness of the didactic and case presentation/discussion, and whether the LC adequately addressed racial and social equity. This survey also included open-ended questions regarding suggestions for improvement (generally and regarding racial and social justice, specifically), one takeaway from the LC session, topics/ideas for future sessions, and remaining questions about the topic (very few participants answered this last question). All responses were recorded anonymously.

**Table 3:** Aggregated responses to quantitative feedback items.

**Variable**	**Wave 1**		**Wave 2**		**Community X**	
	***N***	**Mean (SD) or** ***n*** **(%)**	***N***	**Mean (SD) or** ***n*** **(%)**	***N***	**Mean (SD) or** ***n*** **(%)**
Perceived helpfulness (1 = not at all helpful, 5 = extremely helpful)	78	4.42 (0.61)	40	4.25 (0.77)	15	4.07 (0.68)
Perceived relevance (1 = not at all relevant, 5 = extremely relevant)	78	4.36 (0.82)	40	4.48 (0.74)	15	4.13 (0.96)
Usefulness of didactic (1 = not at all useful, 5 = extremely useful)	75	4.56 (0.57)	40	4.25 (0.70)	28^a^	3.82 (0.54)
Usefulness of case presentation or discussion (1 = not at all useful, 5 = extremely useful)^b^	71	4.25 (0.97)	37	4.18 (0.83)	19	4.00 (0.79)
Presentation addressed racial and social equity	78	70 (90%)	40	36 (90%)	NA^c^	NA^c^

^a^The Community X LC on Bipolar Disorder included two didactics and participants could rate the usefulness of both, thus leading to the greater sample size for this item.

^b^If a LC included a case presentation, the feedback questionnaire included an item about the usefulness of the case presentation, while the LCs that did not include a case presentation had an item about the usefulness of the discussion. Very few LCs asked about both case presentations and discussions (*n* = 3) or neither (*n* = 1).

^c^Community X attendees were not asked whether the presentation addressed racial and social equity, as that question was added later in the series.

Table 2 presents attendance (including both HCS staff and community members) and the number of feedback responses provided. Sessions with particularly strong attendance included *De-Escalation Techniques, Co-Occurring Alcohol Use Disorder and Opioid Use Disorder, Motivational Interviewing*, and *Suicide*. The average attendance across LC sessions was 35 (SD = 10) participants in Wave 1, 26 (SD = 11) in Wave 2, and 40 (SD = 15) in Community X. Response rates to feedback surveys were generally low. For example, although 51 individuals attended the Wave 1 LC on De-Escalation, including 43 individuals who were not HCS staff, only 11 responded to feedback surveys.

Given the low number of feedback responses, we report data on LC session helpfulness, relevance, and usefulness by wave (instead of each session) in Table 3. On average, attendees selected responses of 4 or higher on the 5-point response scales, indicating they found the sessions, didactics, and case presentations “very” helpful, relevant, and useful. However, respondents in the Community X series rated the didactic usefulness slightly lower (Mean rating = 3.8/5). In addition, 90% of participants in both Waves 1 and 2 reported that the presentation addressed racial equity and social justice. This question was not asked of Community X participants, as it was added as a required component after their LC series.

Table 4 includes example responses to open-ended questions. Of note, even fewer participants responded to open-ended questions than to items on helpfulness, relevance, and usefulness. Concerning general improvement, participants commonly reported a desire for more case presentations and more time for discussion. There were also suggestions to make presentations more engaging and easier to follow (e.g., speaking more slowly and reducing technical language/jargon). Suggestions to improve racial equity and social justice content were more wide-ranging. Several participants noted that it was difficult to adequately address this complex and nuanced topic in an hour, particularly given competing demands on time. Other suggestions included providing more data on specific topics (e.g., prevalence rates of given mental health concerns and access to care by race/ethnicity) and acknowledging how race/ethnicity and culture might impact certain experiences (e.g., values and beliefs about health). Takeaways from LCs varied, including increased knowledge of prevalence rates, racial and gender disparities, differential diagnosis considerations, skills/tools (e.g., de-escalation scripts), and resources (e.g., coping skill apps). Likewise, attendees suggested a diverse range of topics for future sessions (see Table 4 for examples). Common themes included specific co-occurring disorders, psychotherapeutic modalities, questions regarding medications for OUD, stigma reduction, abstinence vs. harm reduction goals, housing considerations, specific populations, and traumatic experiences.

**Table 4:** Example responses from open-ended feedback questions.

**Question**	**Example responses**
How can we improve future trainings? (*n* = 119)	• More discussion (“More discussion would have been helpful”) • More case presentations • More engaging presentation (“not going [too] fast”; “possible use of media/video”)
Racial equity & social justice improvement (*n* = 90)	• Insufficient time/complex topic (“In only 1 h it is not possible to cover all these bases”) • Include struggles that uniquely impact individuals in minority groups (“pointing out the difference in experiences of racial minorities and majority groups when they are in situations like needing to be de-escalated would have been important and helpful”) • More statistics (“Maybe a graph on the rates of AUD in different racial/ethnic groups”) • Impact of race/ethnicity (“Maybe acknowledge that race/ethnicity and culture impact our values and belief systems, including health topics”) • Disparities in access to care (“Additional details/insight around differences in access to care between demographics”)
One thing you took away from this training? (*n* = 68)	• Complexity of diagnoses (“How complex the diagnosis for PTSD is and how much overlap there is”) • Gender/race disparities (“Disparities between men and women and within different racial/ethnic communities”) • Efficacy of integrative treatment (“Integrative treatment is highly effective–that's a potential game-changer [system-changer]”) • Importance of building rapport
Topics/ideas for future trainings? (*n* = 124)	• Specific co-occurring disorders (“AUD/SUD and ADHD,” “personality disorders and effective treatments,” “schizophrenia and OUD,” “depression related to using,” “bipolar addiction issues”) • Psychotherapy for SUDs (“EMDR/MI/Expressive Art,” “usefulness of psychotherapy as an element of OUD treatment”) • Medications for SUDs (“appropriate evaluating and medication management with polysubstance use disorder,” “Long term side effects of agonist medication – impacts on mental health”) • Reducing stigma (“How to reduce stigma amongst professionals”, “address bias and stigmas and the effect on staff and clients”, “language to reduce stigma”) • Techniques for/conversations around different substance use goals (“I am always interested [to] learn more about helping patients achieve sustained sobriety,” “more strategies to support the conversation around reducing use,” “what are reasonable expectations of relapse within the OUD community,” “not using opiates but continue to use marijuana or other substances”) • Housing considerations (“ways to advocate for low-barrier access to health care and challenges around Homeless shelters for these folks,” “homelessness and housing”) • Specific populations (“addictions in old age”, “youth usage,” “working with sex offenders,” “LGBTQI culturally competent care,” “opioid use disorder and pregnancy”) • Specific traumatic experiences (“human trafficking,” “domestic violence”)

## Discussion

This effort undertaken by the MA site of the HCS demonstrated the feasibility and utility of LC sessions for disseminating information on evidence-based practices to address co-occurring OUD and mental health concerns, with the ultimate goal of mitigating provider-level barriers to integrated care (e.g., lack of knowledge and training about mental health concerns). Notably, this method for disseminating evidence-based practices was implemented on a large scale, involving communities across the state of MA. While coordinating the LCs required significant time and effort from the planning team, this model is likely more efficient than efforts on a smaller scale (e.g., within individual organizations and agencies), given the reach of the LCs. Furthermore, it has been suggested that an LC series might lead to more long-lasting changes in practice than one-time workshops (Jensen-Doss et al., 2020; Gotham et al., 2023).

Frontline workers and providers showed strong interest in various mental health topics and intervention strategies, as evidenced by high attendance, consistent engagement, and positive responses to feedback surveys. Notably, we documented strong attendance without offering continuing education units (CEUs), underscoring general interest in topics offered. During discussions and open-ended responses, participants often reported that they particularly appreciated learning evidence-based strategies that could be acquired and delivered immediately (e.g., breathing techniques for anxiety and scripts for de-escalation). This highlights traditional barriers to mental health care training, which often focus on complex, multiple-component interventions that require more intensive training. Responses to feedback survey questions on future topics also demonstrated ongoing interest in learning about various co-occurring concerns, treatment modalities, and populations. Overall, the interest among community workers and providers shows the need for initiatives like LC sessions to address knowledge gaps in mental health and OUD, particularly ongoing programs that build upon previous sessions.

### Lessons learned and challenges

Despite the demonstrated feasibility and utility of LC sessions to increase knowledge of co-occurring OUD and mental health concerns among frontline service workers and providers, the planning team also encountered several challenges. An overview of these challenges and suggestions for future LCs is presented in Table 5. We also describe these challenges, lessons learned, and suggestions for future LCs below.

**Table 5:** Challenges lesson learned, and suggestions for future learning collaborative.

**Challenge/lesson learned**	**Suggestions for future LCs**
Developing didactics for a highly heterogeneous audience with varied roles, backgrounds, and training levels	• Minimize medical and psychological jargon • Define all key or unfamiliar terms explicitly • Identify a point person familiar with the audience to review content and guide presenters • Include an introductory session to co-occurring disorders and integrated care concepts • Emphasize concrete, actionable take-home strategies usable across scopes of care • Include both pharmacological and non-pharmacological interventions in each session
Value of shared, peer-based learning that was strengthened by diverse professional backgrounds and lived experience	• Intentionally recruit a diverse group of attendees and presenters • Encourage multidisciplinary participation to support nuanced case discussions • Allocate substantial time for discussion (e.g., at least 50% of each session) in addition to didactics
Community case presentations were a strength, but securing case presenters was difficult	• Engage potential case presenters early in planning • Identify cases first and then tailor didactic topics around them • Encourage team-based case presentations to reduce individual burden • Offer additional support (e.g., practice sessions, slide review) for first-time presenters • Prioritize community presenters, when possible, but continue to include case examples from specialists/didactic presenters when needed
High clinical complexity of patients with OUD and co-occurring mental health conditions	• Address the intersection of addiction, serious mental illness, and structural barriers • Focus on simple, low-resource, immediately actionable strategies (e.g., de-escalation scripts, brief breathing techniques) • Emphasize evidence-based approaches that can be learned quickly and implemented immediately • Include sessions explicitly targeting severe mental health issues and crisis management
Challenges integrating racial and social justice equity within brief sessions	• Provide clear guidance to presenters to include disparities, demographics, and context • Add dedicated sessions focused on racial and social justice equity as part of an ongoing LC series (e.g., housing instability, LGBTQI populations, and other structural determinants of health)

An initial challenge for the planning team was in developing didactics appropriate for individuals with highly heterogeneous roles, backgrounds, and experiences (see Table 1). To address this challenge, didactics were designed to minimize medical and psychological jargon, emphasize concrete take-home messages and strategies that could be implemented immediately, regardless of prior training, and provide resources for continued learning. We also ensured discussion of both pharmacological and non-pharmacological (i.e., behavioral) interventions, so that all attendees could acquire skills within their scope of care. Of note, the TTA Management Specialist and nurse practitioner at the MA site reviewed all slides for didactic presentations prior to the LC sessions to ensure content was at the appropriate level for the diverse group of attendees. Our experience underscores the need for a point person familiar with the audience and prior feedback to guide specialists providing didactics. Despite these efforts to target LC sessions to a diverse group of participants, some participants provided suggestions to make sessions even easier to follow (see Table 4). These suggestions indicated that most medical or psychological terms could benefit from definitions and that some participants might benefit from an initial introduction to the concept of co-occurring disorders and integrated care as a part of the series.

While having attendees from diverse backgrounds posed some challenges, this heterogeneous group of participants also strengthened case-based and shared learning by providing various perspectives on treatment and recovery. Most cases presented had complex presentations (e.g., multiple co-occurring substance use and psychiatric disorders) that often intersected with social determinants of health (e.g., housing status). As such, the multidisciplinary group of attendees allowed for a more nuanced and holistic discussion of patient presentation and potential intervention options. Of note, although most attendees were frontline workers and providers, many shared their lived and living experiences with OUD and/or mental health concerns during the LCs, which also strengthened the discussion and connections. It was clear that participants valued this shared, peer-based learning as the discussion and case presentation portion of the LC series was rated as highly useful (see Table 3), and one point of feedback included more time for discussion (see Table 4). As such, we suggest that future LC series that focus on integrated care for OUD and mental health concerns recruit a diverse range of attendees and presenters and ensure adequate time for discussion among attendees (e.g., at least half of the session).

Case-based learning was both a strength of this LC series and a challenge. Participants rated case presentations as “very useful” and often requested more cases when providing feedback (see Table 4), underscoring the value of case examples in fostering bidirectional learning and active engagement among community members. Yet, it was also challenging to secure case presenters, likely due to time constraints on community providers. Surmounting this barrier may require early engagement with potential participants to determine strategies to increase willingness to present. Potential options may include first recruiting an attendee with a case they would like to discuss, then choosing a didactic topic based on that case (rather than recruiting cases for a given topic), and encouraging “teams” to present a case to reduce the time burden on each individual. Additionally, we noted that many community workers and providers lacked experience with case presentations, which may have impacted their comfort and willingness to present a case in this setting. It may be helpful to provide additional support to those interested in presenting a case, such as meetings to practice slides/presentations. Lastly, content specialists who presented didactics (largely clinician-scientists) occasionally provided case presentations when we could not identify community presenters. While it is preferable to have community case presenters to facilitate connections and learning from provider peers, attendees appeared to appreciate any case examples to support deeper learning.

One unexpected challenge was the complexity of mental health concerns that communities working with people with OUD face. This complexity was evident in both the content requested (see Table 4) and the nature of the case presentations. LC sessions on topics addressing acute and severe issues, such as de-escalation and suicidality, were often requested and among the best attended. Case examples highlighted the challenges of implementing high-quality mental health care against a backdrop of multiple, often chronic mental health and health conditions, severe social and environmental stressors, and a lack of support. The level of patient complexity reported by attendees underscores the need for presenters at LCs to focus on simple, actionable techniques that require minimal resources (e.g., de-escalation scripts) and on evidence-based strategies that can be acquired and delivered immediately (e.g., breathing techniques for anxiety). In addition, future LC series should include sessions that focus on topics at the intersection of addiction, severe mental health care, and structural issues (e.g., medication management in unhoused and unstably housed populations, serious mental illness and OUD).

Lastly, although most participants felt that LCs covered racial and social justice equity (see Table 3), feedback surveys also highlighted the difficulty of adequately integrating information on health disparities within a brief session (see Table 4). Responses to feedback items indicated that instructions provided to both didactic and case presenters to include information on health disparities (didactics) and demographics/contextual considerations (case presentations) appeared to be largely successful. Yet covering racial and social justice equity at a deeper, more nuanced level might require dedicated sessions on these topics as part of an ongoing LC series. Indeed, many attendees expressed interest in racial and social justice equity topics for future sessions, such as housing considerations and LGBTQI populations (see Table 4).

## Limitations and future directions

Although this manuscript represented a Community Case Study describing the implementation of LC sessions, several limitations should be considered when drawing conclusions from it. As the LC sessions themselves were not empirically evaluated (in isolation), we did not require responses to post-session surveys, and response rates were low. Additionally, we did not assess changes in knowledge or implementation of evidence-based practices before and after the LC series. Though our approach likely reflects the real-world engagement and implementation of LC sessions, there is a need to systematically evaluate LC sessions focused on evidence-based practices for co-occurring OUD and mental health concerns. We also did not track repeat attendees; therefore, it is unknown whether session-to-session participants were repeat attendees (which would be more consistent with the purpose of an LC) or were unique. Notably, prior research has demonstrated that individuals with a greater baseline knowledge of the LC topic are more likely to engage in LCs actively (Jensen-Doss et al., 2020). It is also important to note that we primarily employed learning sessions, with no other LC components (e.g., quality improvement teams, PDSA) utilized. The HCS had other ongoing support available to community members (e.g., technical assistance), but there is a need to demonstrate the feasibility of ongoing, peer support for quality improvement among our target population. Lastly, the generalizability of this approach to real-world settings beyond the HCS context needs to be examined. The LC sessions were optional and delivered to front-line workers in community settings, thus largely aligning with real-world settings. However, we also had a large team to assist with planning and delivering the LC series.

## Conclusions

Given the high rates of mental health concerns among those with OUD and their associations with risk of overdose, there is an urgent need to address barriers to integrated treatments. LCs have been described as a method for large-scale dissemination of evidence-based practices through case-based and shared learning (Nadeem et al., 2014). Thus, LCs have utility for filling gaps in knowledge about mental health care reported among frontline workers and providers who serve those with OUD (Snell-Rood et al., 2021; Winiker et al., 2023). The LC series on Mental Health and OUD, delivered to community service workers and providers in MA as part of the HCS, demonstrated that combining case-based learning, interactive discussions, and relevant didactics via learning sessions was perceived as helpful, relevant, and useful. Attendees reported working with patients facing severe psychiatric comorbidities and structural barriers to treatment, such as unstable housing. They also expressed interest in a wide range of mental health topics and concrete strategies for patient care. While there is a clear need for a larger workforce of formally trained mental health professionals to address co-occurring mental health concerns among individuals with OUD, the LC sessions were valuable in offering practical strategies that frontline workers and providers with limited training could apply when managing patients with complex presentations. Based on lessons learned and attendee feedback, future LC sessions should integrate case examples, recruit a diverse range of attendees and presenters, emphasize concrete strategies and resources to enhance clinical practice, and focus sessions on complex topics (e.g., at the intersection of addiction, mental health, and structural barriers to care) and health disparities (e.g., managing co-occurring disorders in subpopulations, such as the elderly and LGBTQI individuals).

## Data Availability

The raw data supporting the conclusions of this article will be made available by the authors, without undue reservation.

## References

[B1] Connery H. S. Taghian N. Kim J. Griffin M. Rockett I. R. H. Weiss R. D. . (2019). Suicidal motivations reported by opioid overdose survivors: a cross-sectional study of adults with opioid use disorder. Drug Alcohol Depend. 205:107612. doi: 10.1016/j.drugalcdep.2019.107612 31627077 PMC6929689

[B2] Connery H. S. Weiss R. D. Griffin M. L. Trinh C. D. Kim J. Rockett I. R. H. . (2022). Suicidal motivations among opioid overdose survivors: replication and extension. Drug Alcohol Depend. 235:109437. doi: 10.1016/j.drugalcdep.2022.109437 35427980 PMC9106902

[B3] Duffy F. F. Chung H. Trivedi M. Rae D. S. Regier D. A. Katzelnick D. J. . (2008). Systematic use of patient-rated depression severity monitoring: is it helpful and feasible in clinical psychiatry? Psychiatr. Serv. 59, 1148–1154. doi: 10.1176/ps.2008.59.10.1148 18832500

[B4] Gotham H. J. Paris M. Hoge M. A. (2023). Learning collaboratives: a strategy for quality improvement and implementation in behavioral health. J. Behav. Health Serv. Res. 50, 263–278. doi: 10.1007/s11414-022-09826-z 36539679 PMC9935679

[B5] Gustafson D. H. Quanbeck A. R. Robinson J. M. Ford I. I. J. H. Pulvermacher A. French M. T. . (2013). Which elements of improvement collaboratives are most effective? A cluster-randomized trial. Addiction 108, 1145–1157. doi: 10.1111/add.12117 23316787 PMC3651751

[B6] Hawk K. F. Vaca F. E. D'Onofrio G. (2015). Reducing fatal opioid overdose: prevention, treatment and harm reduction strategies. Yale J. Biol. Med. 88, 235–245. 26339206 PMC4553643

[B7] Jeffries-Baxter R. Burant C. J. Voss J. G. (2024). The influence of trauma history on opiate use disorder in an urban treatment facility in Pennsylvania. Arch. Psychiatr. Nurs. 53, 242–247. doi: 10.1016/j.apnu.2024.06.023 39615941

[B8] Jensen-Doss A. Smith A. M. Walsh L. M. Mora Ringle V. Casline E. Patel Z. . (2020). Preaching to the choir? Predictors of engagement in a community-based learning collaborative. Admin. Policy Ment. Health 47, 279–290. doi: 10.1007/s10488-019-00985-4 31617139

[B9] Jones C. M. McCance-Katz E. F. (2019). Co-occurring substance use and mental disorders among adults with opioid use disorder. Drug Alcohol Depend. 197, 78–82. doi: 10.1016/j.drugalcdep.2018.12.030 30784952

[B10] LoSavio S. T. Dillon K. H. Murphy R. A. Goetz K. Houston F. Resick P. A. (2019). Using a learning collaborative model to disseminate cognitive processing therapy to community-based agencies. Behav. Ther. 50, 36–49. doi: 10.1016/j.beth.2018.03.007 30661565

[B11] Nadeem E. Olin S. S. Hill L. C. Hoagwood K. E. Horwitz S. M. A. (2014). Literature review of learning collaboratives in mental health care: used but untested. Psychiatr. Serv. 65, 1088–1099. doi: 10.1176/appi.ps.201300229 24882560 PMC4226273

[B12] Novak P. Feder K. A. Ali M. M. Chen J. (2019). Behavioral health treatment utilization among individuals with co-occurring opioid use disorder and mental illness: evidence from a national survey. J. Subst. Abuse Treat. 98, 47–52. doi: 10.1016/j.jsat.2018.12.006 30665603 PMC6350939

[B13] Park T. W. Shuey B. Liebschutz J. Cantor J. Anderson T. S. (2024). Treatment approaches for opioid use disorder offered in us substance use treatment facilities. JAMA 332:502–504. doi: 10.1001/jama.2024.11913 38990551 PMC11240225

[B14] Rodríguez M. N. Colgan D. D. Leyde S. Pike K. Merrill J. O. Price C. J. . (2024). Trauma exposure across the lifespan among individuals engaged in treatment with medication for opioid use disorder: differences by gender, PTSD status, and chronic pain. Subst. Abuse Treat. Prev. Policy 19, 1–11. doi: 10.1186/s13011-024-00608-8 38702783 PMC11067259

[B15] Santo T. Campbell G. Gisev N. Martino-Burke D. Wilson J. Colledge-Frisby S. . (2022). Prevalence of mental disorders among people with opioid use disorder: a systematic review and meta-analysis. Drug Alcohol Depend. 238:109551. doi: 10.1016/j.drugalcdep.2022.109551 35797876

[B16] Snell-Rood C. Pollini R. A. Willging C. (2021). Barriers to integrated medication-assisted treatment for rural patients with co-occurring disorders: the gap in managing addiction. Psychiatr. Serv. 72, 935–942. doi: 10.1176/appi.ps.202000312 33530734

[B17] Streck J. M. Parker M. A. Bearnot B. Kalagher K. Sigmon S. C. Goodwin R. D. . (2022). National trends in suicide thoughts and behavior among US adults with opioid use disorder from 2015 to 2020. Subst. Use Misuse 57, 876–885. doi: 10.1080/10826084.2022.2046102 35232317 PMC9084338

[B18] Substance Abuse and Mental Health Services Administration (2021). National Survey of Substance Abuse Treatment Services (N-SSATS): 2020. Rockville, MD: Center for Behavioral Health Statistics and Quality.

[B19] The HEALing Communities Study Consortium (2024). Community-based cluster-randomized trial to reduce opioid overdose deaths. N. Engl. J. Med. 391, 989–1001. doi: 10.1056/NEJMoa2401177 38884347 PMC11761538

[B20] van Draanen J. Tsang C. Mitra S. Phuong V. Murakami A. Karamouzian M. . (2022). Mental disorder and opioid overdose: a systematic review. Soc. Psychiatry Psychiatr. Epidemiol. 57, 647–671. doi: 10.1007/s00127-021-02199-2 34796369 PMC8601097

[B21] Walsh S. L. El-Bassel N. Jackson R. D. Samet J. H. Aggarwal M. Aldridge A. P. . (2020). The HEALing (Helping to End Addiction Long-term SM) communities study: protocol for a cluster randomized trial at the community level to reduce opioid overdose deaths through implementation of an integrated set of evidence-based practices. Drug Alcohol Depend. 217:108335. doi: 10.1016/j.drugalcdep.2020.108335 33248391 PMC7568493

[B22] Winhusen T. Walley A. Fanucchi L. C. Hunt T. Lyons M. Lofwall M. . (2020). The opioid-overdose reduction continuum of care approach (ORCCA): evidence-based practices in the HEALing communities study. Drug Alcohol Depend. 217:108325. doi: 10.1016/j.drugalcdep.2020.108325 33091842 PMC7533113

[B23] Winiker A. K. Heidari O. Pollock S. Sodder S. Tobin K. E. (2023). Barriers to assessing and treating trauma in primary care and opportunities for improvement: perspectives from prescribers of medications for opioid use disorder. Subst. Use Misuse. 58, 1651–1659. doi: 10.1080/10826084.2023.2238301 37495397 PMC10758239

